# Pituitary Adenylate Cyclase-Activating Polypeptide Ameliorates Experimental Acute Ileitis and Extra-Intestinal Sequelae

**DOI:** 10.1371/journal.pone.0108389

**Published:** 2014-09-19

**Authors:** Markus M. Heimesaat, Ildiko R. Dunay, Silvia Schulze, André Fischer, Ursula Grundmann, Marie Alutis, Anja A. Kühl, Andrea Tamas, Gabor Toth, Miklos P. Dunay, Ulf B. Göbel, Dora Reglodi, Stefan Bereswill

**Affiliations:** 1 Department of Microbiology and Hygiene, Charité - University Medicine Berlin, Berlin, Germany; 2 Department of Microbiology and Hygiene, University of Magdeburg, Magdeburg, Germany; 3 Department of Medicine I for Gastroenterology, Infectious Disease and Rheumatology/Research Center ImmunoSciences (RCIS), Charité - University Medicine Berlin, Berlin, Germany; 4 Department of Anatomy, PTE-MTA Lendület PACAP Research Team, University of Pecs, Pecs, Hungary; 5 Department of Medical Chemistry, University of Szeged, Szeged, Hungary; 6 Department and Clinic of Surgery and Ophthalmology, Faculty of Veterinary Medicine, Szent Istvan University Budapest, Budapest, Hungary; University of Hong Kong, Hong Kong

## Abstract

**Background:**

The neuropeptide Pituitary adenylate cyclase-activating polypeptide (PACAP) plays pivotal roles in immunity and inflammation. So far, potential immune-modulatory properties of PACAP have not been investigated in experimental ileitis.

**Methodology/Principal Findings:**

Mice were perorally infected with *Toxoplasma (T.) gondii* to induce acute ileitis (day 0) and treated daily with synthetic PACAP38 from day 1 to 6 post infection (p.i.; prophylaxis) or from day 4 to 6 p.i. (therapy). Whereas placebo-treated control mice suffered from acute ileitis at day 7 p.i. and succumbed to infection, intestinal immunopathology was ameliorated following PACAP prophylaxis. PACAP-treated mice exhibited increased abundance of small intestinal FOXP3+ cells, but lower numbers of ileal T lymphocytes, neutrophils, monocytes and macrophages, which was accompanied by less ileal expression of pro-inflammatory cytokines such as IL-23p19, IL-22, IFN-γ, and MCP-1. Furthermore, PACAP-treated mice displayed higher anti-inflammatory IL-4 concentrations in mesenteric lymph nodes and liver and higher systemic anti-inflammatory IL-10 levels in spleen and serum as compared to control animals at day 7 p.i. Remarkably, PACAP-mediated anti-inflammatory effects could also be observed in extra-intestinal compartments as indicated by reduced pro-inflammatory mediator levels in spleen (TNF-α, nitric oxide) and liver (TNF-α, IFN-γ, MCP-1, IL-6) and less severe histopathological sequelae in lungs and kidneys following prophylactic PACAP treatment. Strikingly, PACAP prolonged survival of *T. gondii* infected mice in a time-of-treatment dependent manner.

**Conclusion/Significance:**

Synthetic PACAP ameliorates acute small intestinal inflammation and extra-intestinal sequelae by down-regulating Th1-type immunopathology, reducing oxidative stress and up-regulating anti-inflammatory cytokine responses. These findings provide novel potential treatment options of inflammatory bowel diseases.

## Introduction

Pituitary adenylate cyclase-activating polypeptide (PACAP) was first identified as a hypothalamic neuropeptide stimulating adenylate cyclase activity in the pituitary gland [1]. The peptide is a member of the vasoactive intestinal peptide (VIP)/secretin/glucagon family and shares 68% homology with VIP. Following alternative splicing from its pre-pro precursor, PACAP is present in two biologically active amidated forms, namely PACAP27 and PACAP38 [1], [2]. Besides the central nervous system, PACAP is widely expressed in peripheral organs of the endocrine, reproductive, respiratory, and digestive system as well as in lymphoid organs including immune cells [2]. PACAP exerts its immune-modulatory functions following binding on three receptors. Whereas both, VIP and PACAP, can bind to VPAC1 and VPAC2 receptors present on immune cells such as lymphocytes and macrophages, PAC1 comprises a receptor specific for PACAP, which is expressed by macrophages but not lymphocytes [3]–[5]. The potent anti-inflammatory properties of exogenous PACAP have been demonstrated in experimental models of human arthritis [6] and encephalomyelitis [7], for instance, but data regarding beneficial effects exerted by synthetic PACAP in the gut are limited. PACAP^−/−^ mice subjected to dextran sodium sulfate (DSS) displayed more severe acute colitis as compared to wildtype control animals [8], [9]. In experimental trinitrobenzene sulfonic acid (TNBS) induced colitis, VIP exerted prophylactic and therapeutic effects [10], hence providing evidence for anti-inflammatory effects of PACAP and VIP in the intestinal tract.

Inflammatory bowel diseases (IBD) such as ulcerative colitis or Crohn's disease (also termed ileitis terminalis) are of multi-factorial etiology and characterized by chronic intestinal inflammation with acute episodes [11]–[13]. The vast majority of intestinal inflammation studies have been performed in colitis models, whereas small intestinal inflammation *in vivo* models are scarce.

Within one week following peroral infection with 100 cysts of the parasite *Toxoplasma (T.) gondii*, susceptible mice develop severe small intestinal inflammation (pan-ileitis) and succumb to infection within seven to ten days. The underlying immunopathology is characterized by an IL-23-induced Th1-type immune response with a subsequent excessive release of pro-inflammatory mediators such as IL-22, IL-18, IL-12, IFN-γ, TNF-α, and nitric oxide (NO) [14]–[19], whereas *T. gondii* induced counter-regulatory cytokines include IL-10 [14], [20]. Furthermore, inflammation is accompanied by distinct shifts in commensal ileal microbiota composition. Gram-negative species such as *E. coli* and *Bacteroides/Prevotella* species overgrow the inflamed ileal lumen during ileitis development [21], and the inflammatory scenario is further perpetuated by TLR-4-dependent signaling of lipopolysaccharide (LPS) derived from intestinal Gram-negative commensals [22], [23]. Taken together, the Th1-type immunopathology in the *T. gondii*-mediated ileitis model resembles immunopathological key features of acute episodes in Crohn's disease [24], [25]. Given that the immune-modulatory properties of PACAP have never been studied in small intestinal inflammation, we assessed potential beneficial effects of the synthetic compound in the *T. gondii*-induced acute ileitis model. Results reveal distinct time-of-treatment-dependent anti-inflammatory properties of synthetic PACAP. Remarkably, PACAP-mediated amelioration of small intestinal immunopathology was accompanied by systemic anti-inflammatory effects in extra-intestinal compartments such as blood, spleen, liver, kidney, and lung.

## Methods

### Mice, ethical statement

C57BL/6 (wildtype) mice were bred and housed under specific pathogen-free (SPF) conditions in the Forschungseinrichtung für Experimentelle Medizin (FEM, Charité – University Medicine Berlin, Berlin, Germany). All animal experiments were conducted according to the European Guidelines for animal welfare (2010/63/EU) with approval of the commission for animal experiments headed by the “Landesamt für Gesundheit und Soziales” (LaGeSo, Berlin, registration number G0145/10). Animal welfare was monitored twice daily by assessment of clinical conditions and weight loss of mice. Mice suffering from weight loss >20% were humanely euthanized by isofluran treatment (Abbott, Germany) in accordance with the guidelines of the local commission for animal experiments headed by the “Landesamt für Gesundheit und Soziales”.

### Induction of acute ileitis

For induction of acute ileitis, 3 months old female mice were infected perorally by gavage with 100 *T. gondii* cysts (ME49 strain) from homogenized brains of intraperitoneally infected NMRI mice in a volume of 0.3 ml phosphate-buffered saline (PBS), as described previously [21], [22], [26].

### Treatment

PACAP38 was synthesized at the Department of Medical Chemistry, University of Szeged (Hungary), dissolved in PBS and administered to mice with a daily dose of 1.5 mg per kg body weight [9]. Mice received the PACAP or PBS (serving as placebo control, PLC) intraperitoneally (i.p.) in a 0.3 ml volume once daily starting one day (prophylactic treatment regimen) or four days (therapeutic treatment regimen) following *T. gondii* infection until day 6 post infection. Age- and sex-matched naïve mice served as negative controls. A potential anti-bacterial effect of the PACAP solution was excluded as described previously [27].

### Sampling procedures, determination of small intestinal shortening

Mice were sacrificed by isoflurane (Abbott, Wiesbaden, Germany) seven days after infection. Cardiac blood and tissue samples from lungs, liver, spleen, kidneys, mesenteric lymph nodes (MLNs), and terminal ileum were removed under sterile conditions. Intestinal samples were collected for histopathological, immunohistochemical, and immunological analyses. Small intestinal lengths were determined by measuring the distance from the duodenum leaving the stomach to the ileal-caecal valve by a ruler. The relative shortening of the small intestine was calculated by dividing the difference of the mean length of small intestine from age- and sex-matched naïve control mice minus the length of infected mice at day 7 p.i., and then multiplied by 100 over the mean length of naïve control mice small intestines (relative shortening in length = (mean d0−d7 p.i.)×100/mean d0). Results were expressed as % shortage.

### Histopathology and determination of parasite loads

Histopathological changes were determined in samples derived from terminal ileum, lungs, and kidneys that were immediately fixed in 5% formalin and embedded in paraffin. Sections (5 µm) were stained with hematoxylin and eosin (H&E) and examined by light microscopy (magnification 100× and 400×). Histopathological changes in the small intestine were quantitatively assessed applying a standardized histopathological scoring system (ranging from 0 to 6) for blinded duplicate evaluation as described in detail earlier [21]. *T. gondii* DNA was quantified in ileal biospies as described earlier and expressed in pg [19].

### Immunohistochemistry

*In situ* immunohistochemical analysis of ileum paraffin sections were performed as described previously [28]. Primary antibodies against CD3 (#N1580, Dako, Denmark, dilution 1∶10), FOXP-3 (FJK-16s, eBioscience, 1∶100), myeloperoxidase-7 (MPO-7, # A0398, Dako, 1∶500), and F4/80 (#14-4801, clone BM8, eBioscience, 1∶50) were used. For each animal, the average number of positively stained cells within at least six independent high power fields (HPF, 0.287 mm^2^; 400× magnification) was determined microscopically by two independent double-blinded investigators.

### Real-time PCR

RNA was isolated from snap frozen *ex vivo* ileum biopsies, reverse transcribed and analyzed as described previously [19]. Murine IL-23p19 and IL-22 mRNA expressions were detected and analyzed using Light Cycler Data Analysis Software (Roche). Expression levels were calculated relative to the HPRT expression and indicated as “Arbitrary Units”.

### Detection of cytokine secretion in *ex vivo* biopsies

Ileal biopsies were cut longitudinally, washed with PBS and strips of 1 cm^2^, MLNs, liver or spleen placed in 24-flat-bottom well culture plates (Nunc, Wiesbaden, Germany) containing 500 µl serum-free RPMI 1640 medium supplemented with penicillin (100 U/ml) and streptomycin (100 µg/ml; PAA Laboratories). After 18 h at 37°C, culture supernatants were tested for IFN-γ, TNF-α, MCP-1, IL-6, and IL-10 concentrations by the Mouse Inflammation Cytometric Bead Assay (CBA; BD Biosciences) in a BD FACSCanto II flow cytometer (BD Biosciences). IL-4 concentrations were measured by ELISA (BD Biosciences), and nitric oxide (NO) was determined by Griess reaction as described earlier [21].

### Statistical analysis

Mean values, medians, standard deviations, standard errors of the mean, and levels of significance were determined using appropriate tests as indicated (Mann-Whitney-U test) or the log-rank test for Kaplan-Meier analysis of survival. Two-sided probability (p) values ≤0.05 were considered significant. All experiments were repeated at least twice.

## Results

### PACAP treatment prolongs survival in acute ileitis

**Figure 1:**
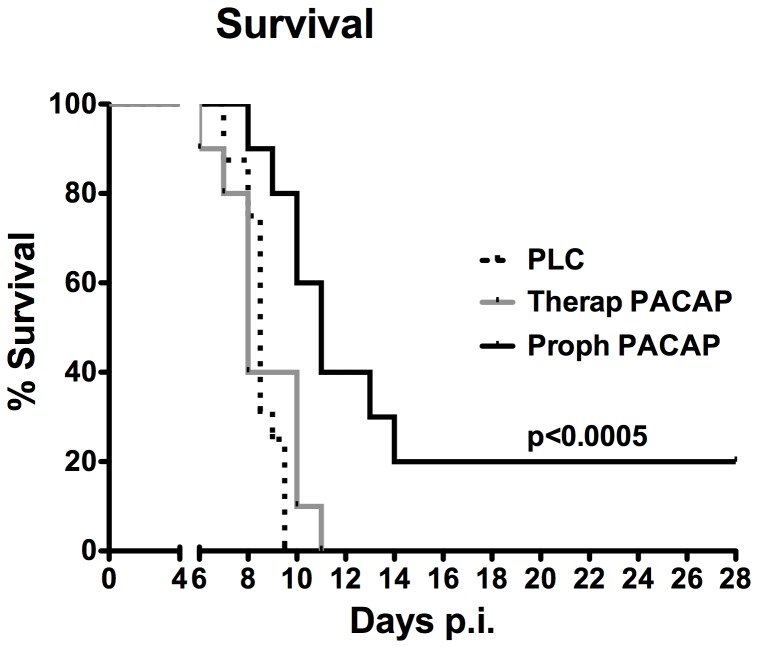
Survival rates of PACAP treated mice. Ileitis was induced by peroral infection of mice with *T. gondii* at day 0 as described in methods. Survival of animals following prophylactic (Proph, black line; n = 10) or therapeutic (Therap, gray line; n = 10) PACAP treatment as compared to placebo controls (PLC, dotted line; n = 16) was monitored twice daily until day 28 post infection (p.i.). Significance levels (as compared to respective groups) were determined by Kaplan-Meier analysis. Data are pooled from three independent experiments.

Following ileitis induction (day 0) *T. gondii* infected mice were treated with synthetic PACAP (1.5 mg per kg body weight per day) via the intraperitoneal route, either from day 1 until day 6 (prophylactic regimen) post infection (p.i.) or starting on day 4, when first histopathological ileum mucosal changes can be observed [21], until day 6 following ileitis induction (therapeutic treatment) and compared to control animals. Given that the outcome of PLC treated mice was comparable, irrespective whether the PLC was administered starting by day 1 or 4 p.i., derived data were respectively pooled to one PLC treated group as indicated. Whereas all PLC treated mice had died by day 9.5 p.i., 80% of mice with PACAP prophylaxis and 40% of mice in the therapeutic group survived the acute phase of inflammation (**Fig. 1**). All mice subjected to short-term PACAP treatment, however, died until day 11 p.i., whereas by that time 40% of mice from the prophylaxis group were still alive and 20% survived the end of the experiment (28 days p.i.; p<0.005 vs. PLC, Kaplan Meier analysis) (**Fig. 1**). Thus, PACAP prolongs survival of acute ileitis when given prophylactically (i.e. before the onset of histopathological ileal changes).

### PACAP ameliorates acute small intestinal inflammation

**Figure 2:**
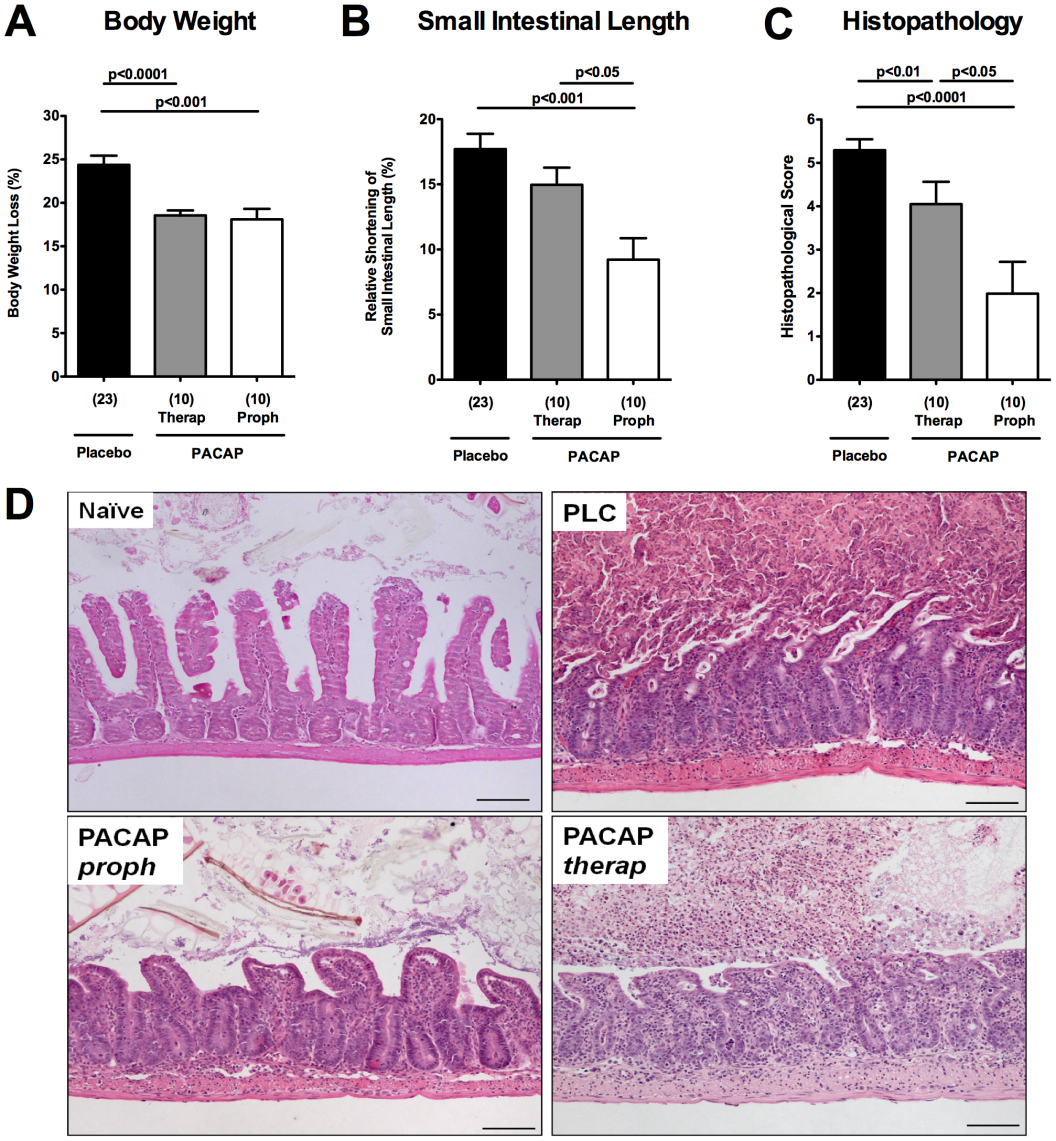
Amelioration of acute ileitis in PACAP-treated mice. Ileitis was induced by peroral infection of mice with *T. gondii* at day 0. (A) Relative body weight loss of mice (in %), (B) relative small intestinal shortening (in %), and (C) histopathological scores of the terminal ileum following PACAP prophylactic treatment (Proph, white bars), PACAP therapy (Therap, gray bars) or placebo application (black bar) at day 7 post infection. Numbers of analyzed animals are given in parentheses. Mean values, standard errors of the mean, and significance levels as determined by the Mann-Whitney-U test are indicated. Data are pooled from three independent experiments. (D) Representative photomicrographs (100× magnification; scale bar 100 µm) of histopathological changes in ileal paraffin sections from three independent experiments are shown. Naïve, uninfected mice served as controls.

Within 7 days p.i., PLC-treated control animals developed wasting disease and lost up to 25% of their initial body weight whereas mice from either PACAP group displayed significantly less weight loss (approximately 18%; p<0.001 vs. PLC; **Fig. 2A**) indicative for less severe disease. Given that small intestinal inflammation is accompanied by a significant shortening of the proximal intestinal tract [21], [29], [30], we determined the lengths of the small intestines between PACAP- and PLC-treated mice. Mice from the PACAP-prophylaxis group exhibited less shrinkage of the small intestines as compared to short-term PACAP treated and control mice (p<0.05 and p<0.001, respectively; **Fig. 2B**).

We next assessed histopathological changes in the ileal mucosa. Whereas at day 7 p.i. PLC mice displayed severe ileal necrosis (**Fig. 2C, D**), mice with therapeutic PACAP treatment, however, exhibited rather moderate ileal inflammation without necrosis (p<0.01 vs PLC). Remarkably, mice subjected to PACAP prophylaxis displayed only mild signs of inflammation as indicated by edema of the villi and discrete cell-free exudate into the lumen, but intact epithelium (p<0.0001 vs PLC; **Fig. 2C, D**). To examine whether differences in ileal *T. gondii* loads might have contributed to the observed beneficial PACAP effects we assessed *T. gondii* DNA concentrations in the respective groups. Notably, the amount of *T. gondii* DNA in the ileum did not differ between the respective PACAP-treated and control mice at day 7 p.i. (**Fig. S1**). Furthermore, PACAP did not exert any direct anti-bacterial effects *in vitro* (not shown). Hence, PACAP-induced changes in the commensal microbiota composition which in turn might impact the disease outcome could be excluded.

Taken together, acute ileitis was ameliorated following PACAP treatment in a time-of-treatment dependent manner as indicated by significantly higher survival rates, better clinical as well as histopathological outcome in prophylactically PACAP treated mice.

### Diminished local intestinal inflammation following PACAP application

**Figure 3:**
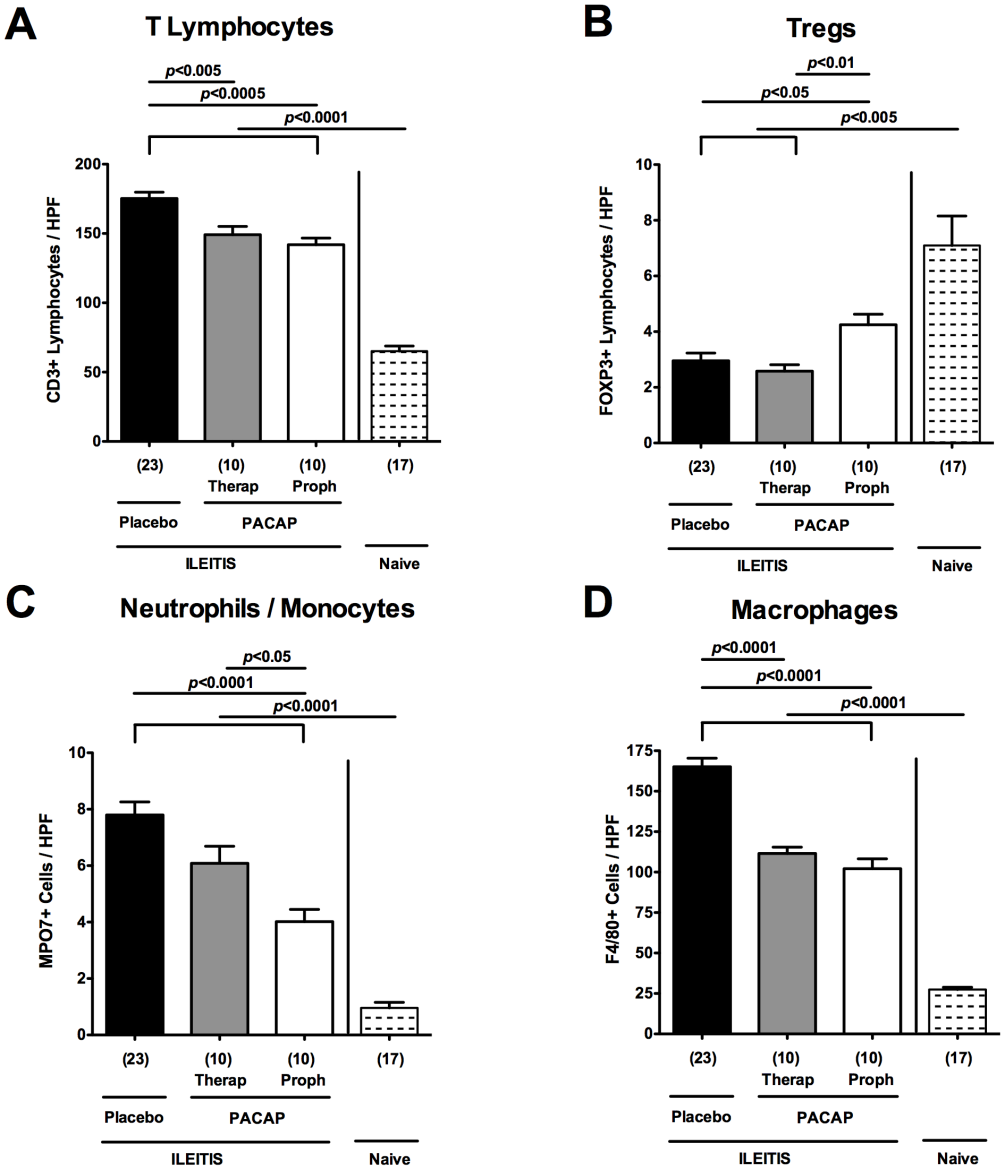
Intestinal immune cell responses following ileitis induction in PACAP-treated mice. Ileitis was induced by peroral infection of mice with *T. gondii* at day 0. The average number of cells positive for (A) CD3 (T lymphocytes), (B) FOXP3 (regulatory T cells, Tregs), (C) MPO7 (myeloperoxidase-7, neutrophils and monocytes), and (D) F4/80 (macrophages) from at least six high power fields (HPF, 400× magnification) per animal were determined microscopically in immunohistochemically stained ileum sections derived from mice following PACAP prophylactic treatment (Proph, white bars), PACAP therapy (Therap, gray bars), or placebo application (black bar) at day 7 post infection as compared to naïve controls (lined bars). Numbers of analyzed animals are given in parentheses. Mean values, standard errors of the mean, and significance levels as determined by the Mann-Whitney-U test are indicated. Data are pooled from three independent experiments.

We next quantitatively assessed the influx of distinct immune cell populations into the ileal mucosa of PACAP treated mice by immunohistochemical staining of ileal paraffin sections. Given that T cells are the major driving forces of *T. gondii*-induced acute ileitis counteracted by IL-10 producing cells such as regulatory T cells (Tregs) [14], [20], we quantified ileal CD3+ and FOXP3+ cells. Seven days following *T. gondii* infection, CD3+ T lymphocytes increased multifold, but were less abundant in mice of either PACAP treatment group as compared to PLC-treated animals (p<0.005-0.0005; **Fig. 3A**). Conversely, FOXP3+ Treg numbers decreased upon *T. gondii* infection. Following PACAP prophylaxis, however, ileal FOXP3+ cell numbers were approximately 50% higher as compared to PLC-treated control and short-term PACAP-treated mice (p<0.05 and p<0.01, respectively; **Fig. 3B**). Given that neutrophils, monocytes, and macrophages are pivotal effector immune cells during acute ileitis development exerting oxidative stress to the intestinal epithelium [25], we investigated the recruitment of MPO7+ and F4/80+ cells in the small intestinal lamina propria. Ileal numbers of neutrophils, monocytes, and macrophages increased multifold upon ileitis development (p<0.0001 vs. naive controls; **Fig. 3C, D**). At day 7 p.i., however, mice subjected to prophylactic PACAP treatment exhibited lower ileal MPO7+ cells as compared to mice with therapeutic PACAP or PLC treatment (p<0.05 and p<0.0001, respectively; **Fig. 3C, D**). Notably, ileal F4/80+ cell numbers decreased in mice of both PACAP groups as compared to PLC-treated control animals at day 7 p.i. (p<0.0001; **Fig. 3C, D**).

**Figure 4:**
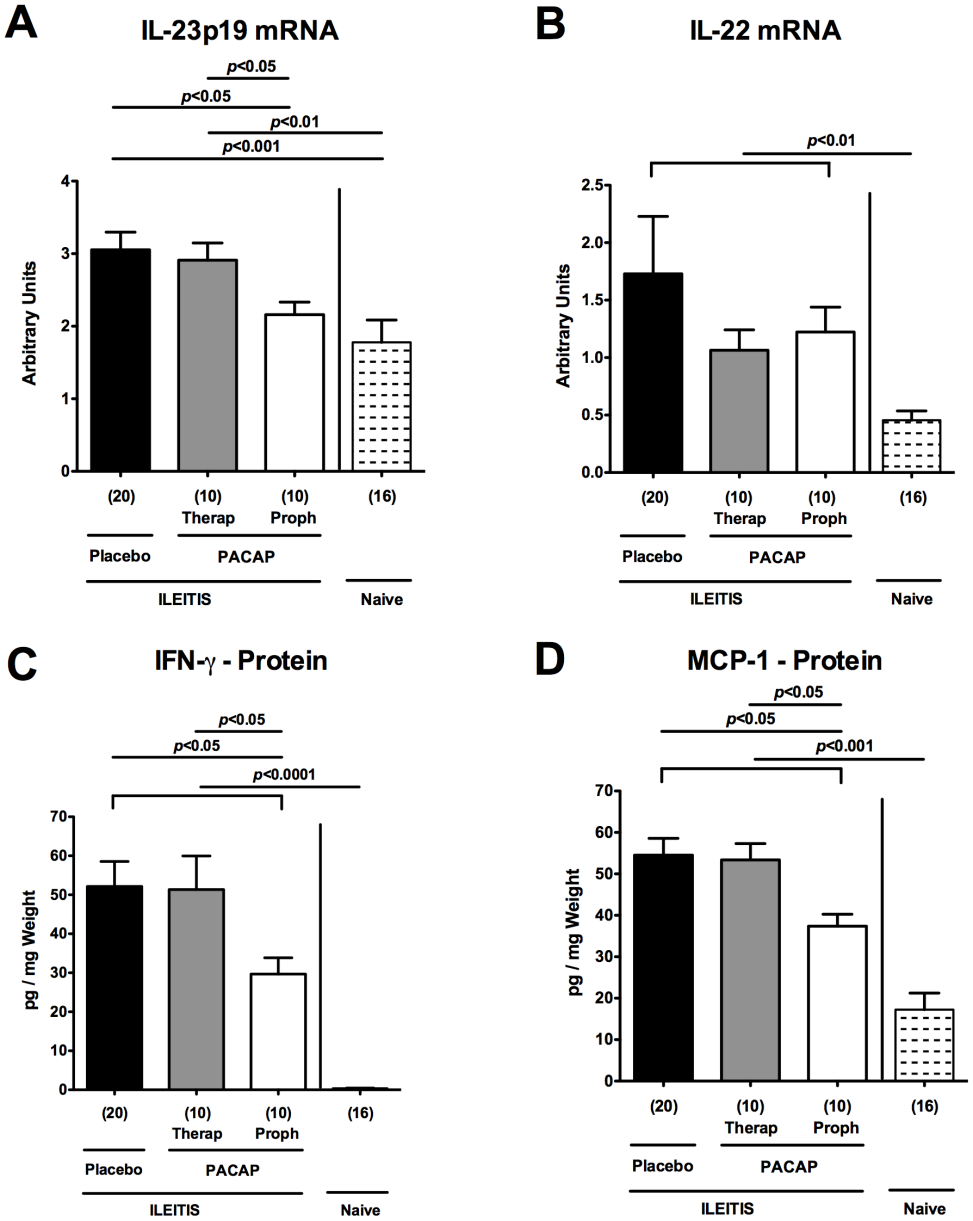
Pro-inflammatory cytokine responses in ilea of PACAP-treated mice. Ileitis was induced by peroral infection of mice with *T. gondii* at day 0. (A) IL-23p19 and (B) IL-22 mRNA expression levels (by RT-PCR) as well as (C) IFN-γ and (D) MCP-1 protein concentrations (by CBA) were determined in *ex vivo* ileum biopsies obtained from mice following PACAP prophylactic treatment (Proph, white bars), PACAP therapy (Therap, gray bars), or placebo application (black bars) at day 7 post infection as compared to naïve animals (lined bars). mRNA expression levels are expressed as fold changes relative to HPRT mRNA expression (Arbitrary Units). Numbers of analyzed animals are given in parentheses. Mean values, standard errors of the mean, and significance levels as determined by the Mann-Whitney-U test are indicated. Data are pooled from three independent experiments.

**Figure 5:**
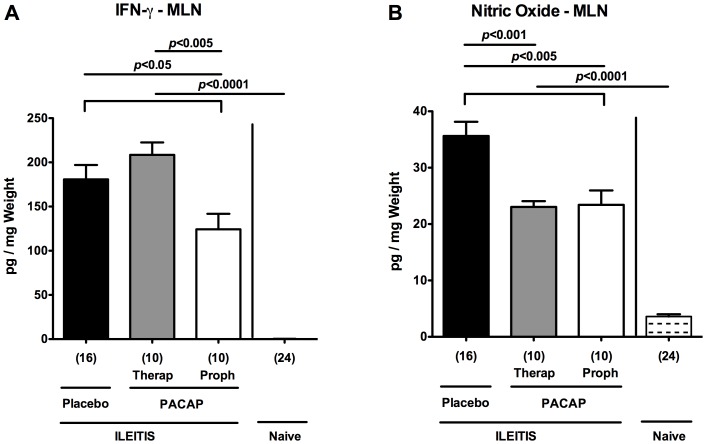
Pro-inflammatory cytokine responses in mesenteric lymph nodes of PACAP-treated mice. Ileitis was induced by peroral infection of mice with *T. gondii* at day 0. (A) IFN-γ and (B) nitric oxide secretion were determined in overnight cultures of *ex vivo* biopsies derived from mesenteric lymphnodes (MLNs) of mice following PACAP PACAP prophylactic treatment (Proph, white bars), PACAP therapy (Therap, gray bars), or placebo application (black bars) at day 7 post infection as compared to naïve animals (lined bars). Numbers of analyzed animals are given in parentheses. Mean values, standard errors of the mean, and significance levels as determined by the Mann-Whitney-U test are indicated. Data are pooled from three independent experiments.

To further unravel immune-modulatory properties of PACAP treatment we determined mRNA expression levels of pro-inflammatory cytokines in *ex vivo* ileal biopsies. IL-23p19 and IL-22 mRNA were both up-regulated in the ileum 7 days following *T. gondii* infection (p<0.001 and p<0.01, respectively; **Fig. 4A, B**). Upon PACAP prophylaxis, however, ileal IL-23p19, acting as a main inducing factor within the Th1 inflammatory cascade [19], [25], was down-regulated (p<0.05; **Fig. 4A**), whereas IL-22 mRNA levels tended to be lower as compared to PLC treated mice at day 7 p.i. (n.s.; **Fig. 4B**). On protein level, ileal IFN-γ and MCP-1 concentrations were lower in *ex vivo* ileum biopsies following PACAP prophylaxis as compared to PLC-treated mice (p<0.05; **Fig. 4C, D** **;** **Table 1**), whereas local TNF-α expression levels did not differ at day 7 p.i. (**Table 1**). The diminished intestinal pro-inflammatory responses upon PACAP application was further underlined by lower IFN-γ and NO protein concentrations measured in MLNs derived from mice subjected to PACAP prophylaxis as compared to control animals at day 7 p.i. (p<0.05 and p<0.005, respectively; **Fig. 5** **;** **Table 1**). Notably, NO levels were also decreased in MLNs following therapeutic PACAP as compared to PLC treatment (p<0.001; **Fig. 5B**). Taken together, PACAP treatment ameliorates *T. gondii* induced small intestinal Th1-type immune responses.

**Table 1:** Summary of pro- and anti-inflammatory mediator regulation following prophylactic PACAP versus placebo treatment at day 7 p.i.

PACAP (proph.) Vs. PLC	IFN-γ	TNF-α	MCP-1	IL-6	NO	IL-4	IL-10
Ileum	⇓	⇒	⇓	⇒	⇒	⇒	⇒
MLN	⇓	⇒	⇒	⇒	⇓	⇑	⇒
Liver	⇓	⇓	⇓	⇓	⇒	⇑	⇒
Spleen	⇒	⇓	⇒	⇒	⇒	⇒	⇑
Serum	⇒	⇒	⇒	⇒	⇒	⇒	⇑

**⇓**: lower **⇒**: comparable **⇑**: higher levels.

### Diminished extra-intestinal pro-inflammatory immune responses following PACAP application in acute ileitis

**Figure 6:**
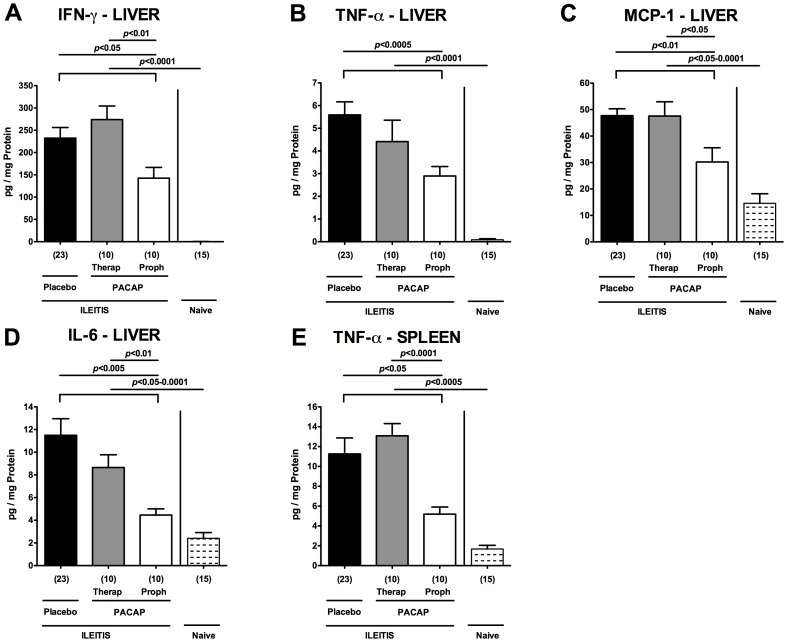
Pro-inflammatory cytokine responses in extra-intestinal compartments of PACAP-treated mice. Ileitis was induced by peroral infection of mice with *T. gondii* at day 0. Hepatic (A) IFN-γ, (B) TNF-α, (C) MCP-1, (D) IL-6, and (E) splenic TNF-α secretion were determined in overnight cultures of *ex vivo* biopsies derived from respective organs of mice following PACAP PACAP prophylactic treatment (Proph, white bars), PACAP therapy (Therap, gray bars), or placebo application (black bars) at day 7 post infection as compared to naïve animals (lined bars). Numbers of analyzed animals are given in parentheses. Mean values, standard errors of the mean, and significance levels as determined by the Mann-Whitney-U test are indicated. Data are pooled from three independent experiments.

In the following we investigated whether potential extra-intestinal *T. gondii*-induced sequelae were diminished following PACAP treatment. To address this, we measured pro-inflammatory cytokines in *ex vivo* biopsies derived from liver and kidneys of infected and naïve mice. Upon *T. gondii* infection, hepatic IFN-γ, TNF-α, MCP-1, and IL-6 protein levels increased multi-fold (p<0.05-0.0001 vs naïve mice; **Fig. 6A–D**). Respective pro-inflammatory cytokine concentrations, however, were lower in livers following PACAP prophylaxis as compared to controls at day 7 p.i. (p<0.05-0.0005; **Fig. 6A–D** **;** **Table 1**). In addition, mice from the PACAP prophylaxis group exhibited lower splenic TNF-α levels as compared to PLC-treated controls at day 7 p.i. (p<0.05; **Fig. 6E** **;** **Table 1**). Hence, PACAP dampens pro-inflammatory responses in *T. gondii* induced acute ileitis not only locally but also systemically affecting extra-intestinal compartments.

### Up-regulated anti-inflammatory immune responses following PACAP application in acute ileitis

**Figure 7:**
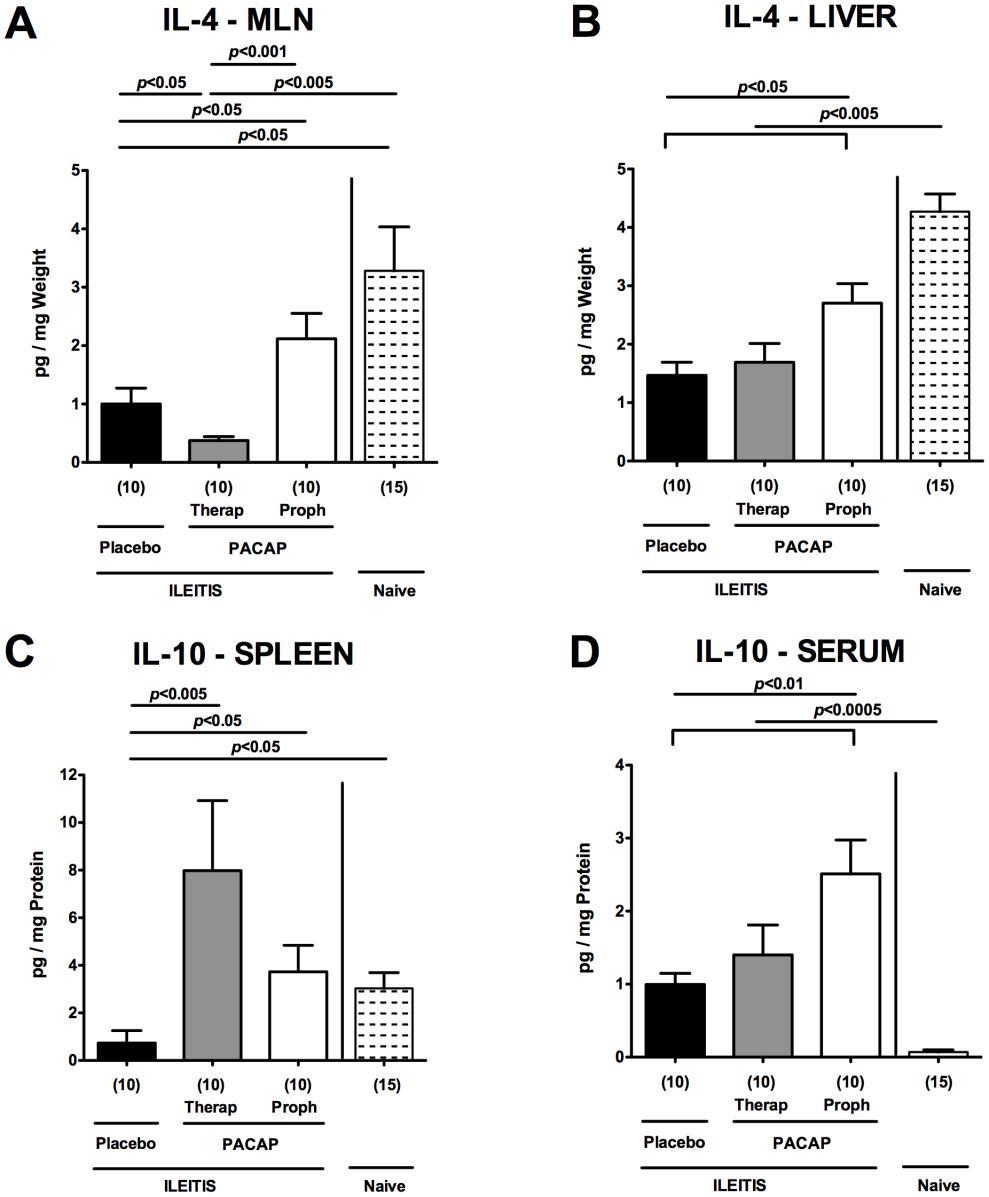
Anti-inflammatory cytokine responses in extra-intestinal compartments of PACAP-treated mice. Ileitis was induced by peroral infection of mice with *T. gondii* at day 0. IL-4 protein levels were determined in *ex vivo* biopsies of (A) mesenteric lymph nodes (MLN) and (B) liver, and IL-10 concentrations measured in (C) spleen and (D) serum of mice following PACAP prophylactic treatment (Proph, white bars), PACAP therapy (Therap, gray bars), or placebo application (black bars) at day 7 post infection as compared to naïve animals (lined bars). Numbers of analyzed animals are given in parentheses. Mean values, standard errors of the mean, and significance levels as determined by the Mann-Whitney-U test are indicated. Data are pooled from three independent experiments.

We next investigated whether PACAP-induced anti-inflammatory responses in the small intestine and extra-intestinal compartments might have contributed to the improved outcome following treatment of mice with the synthetic compound. Following PACAP prophylaxis, mice displayed higher IL-4 protein levels in *ex vivo* biopsies derived from MLNs and livers at day 7 p.i. as compared to PLC treated mice (p<0.05; **Fig. 7A, B** **;** **Table 1**). Remarkably, also systemic anti-inflammatory responses were up-regulated in mice subjected to PACAP prophylaxis as indicated by higher IL-10 protein levels in spleen and serum following long-term PACAP as compared to PLC treatment at day 7 p.i. (p<0.05 and p<0.01, respectively; **Fig. 7C, D** **;** **Table 1**). In addition, mice with therapeutic PACAP treatment displayed higher splenic IL-10 concentrations as comparted to PLC-treated control animals (p<0.005; **Fig. 7C**).

**Figure 8:**
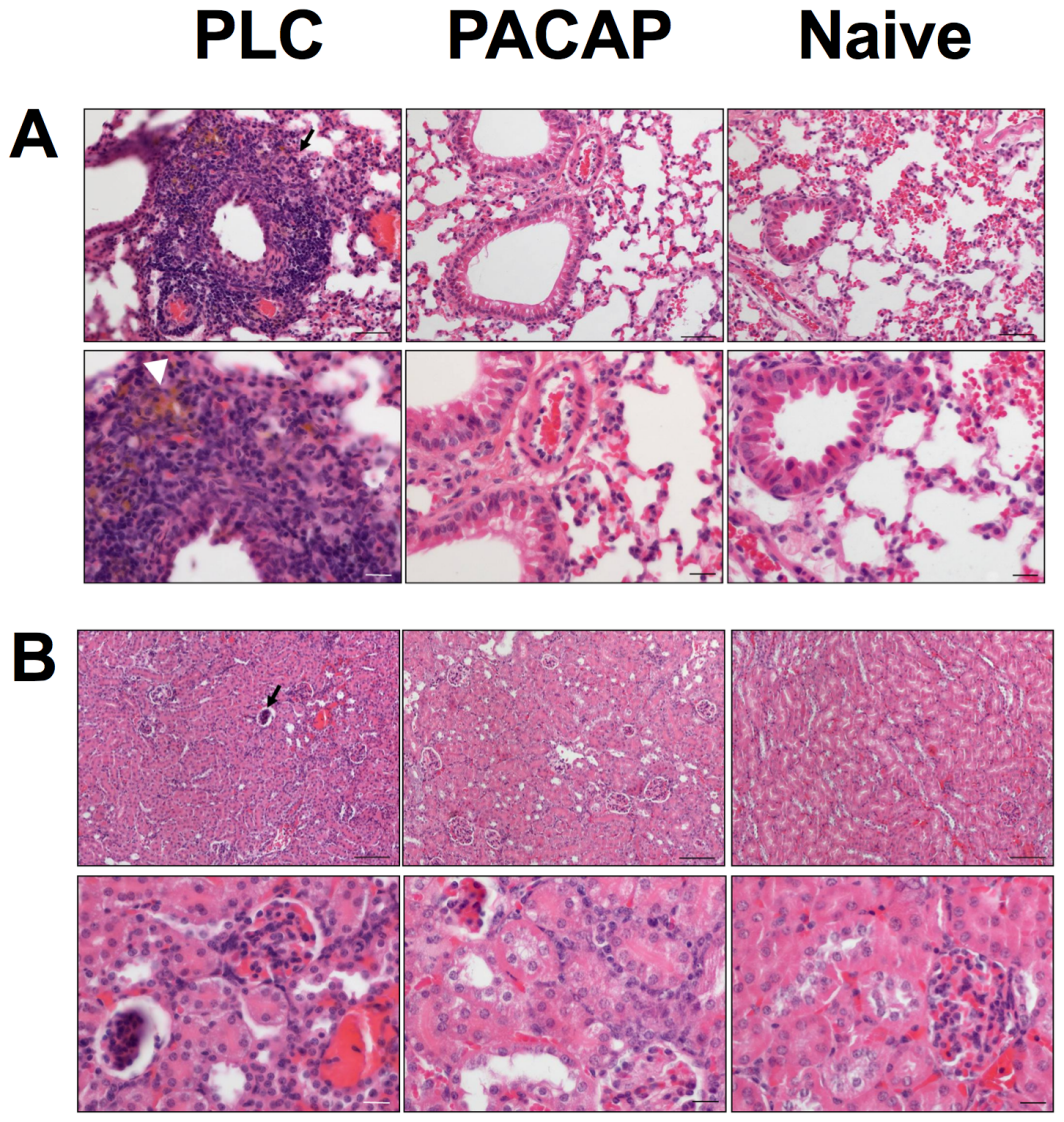
Less severe extra-intestinal histopathology following PACAP prophylaxis. Paraffin section of (A) lung and (B) kidney samples were obtained from naïve animals (right panel) and from mice following PACAP prophylactic treatment (PACAP, middle panel) and compared to placebo controls (PLC, left panel) at day 7 post infection. Representative photomicrographs of H&E stained paraffin sections (upper panel: 100× magnification; scale bar 100 µm; lower panel: 400× magnification; scale bar 20 µm) from three independent experiments are shown. White arrow head points towards hemosiderin deposit (in A), black arrows indicate an inflammatory peri-bronchial cuff (in A) and a pyknotic, degenerated, apoptotic glomerulum (in B).

Significant beneficial extra-intestinal effects exerted by prophylactic PACAP application were further supported by examination of H&E-stained paraffin sections taken from lungs and kidneys at day 7 p.i. (**Fig. 8**). Whereas lungs of PLC-treated mice suffering from acute ileitis displayed significant peri-bronchial cuffs of inflammatory cells and hemosiderin deposits therein, lungs following PACAP prophylaxis appeared similar to those of uninfected mice (**Fig. 8A**). Furthermore, pyknotic, degenerating and finally apoptotic glomeruli were observed in kidneys of PLC-, but not PACAP-treated mice at day 7 p.i. (**Fig. 8B**). Noteworthy, this is the first analysis of inflammatory sequelae affecting lungs and kidneys in the *T. gondii* induced acute ileitis model.

Taken together, *T. gondii* infected mice subjected to a prophylactic PACAP regimen did not only display less pro-inflammatory Th1-type immunopathology within the ileum, but also exerted beneficial extra-intestinal and systemic effects as indicated by down-regulated pro- and up-regulated anti-inflammatory responses.

## Discussion

A plethora of previous *in vitro* and *in vivo* studies revealed that the neuropeptide PACAP acts as a pleiotropic immune-modulator [31]. For the first time we investigated the immune-modulatory properties of prophylactic and therapeutic synthetic PACAP administration in murine *T. gondii* induced acute ileitis. Importantly, PACAP did not exhibit any anti-bacterial effects *in vitro*, which might have directly interfered with intestinal microbiota composition subsequently impacting disease outcome. Given that anti-parasitic effects of PACAP and VIP against *Trypanosoma brucei* were described previously [32], [33], we started prophylactic PACAP treatment one day following *T. gondii* infection in order to minimize direct interference of the compound with the parasite thereby counteracting the induction of inflammation. Notably, ileal *T. gondii* DNA loads did not differ between the respective treatment groups at day 7 p.i. Whereas control mice suffered from acute pan-ileitis with necroses at day 7 p.i. and succumbed to infection, PACAP treated mice were protected from ileal immunopathology in a time-of-treatment dependent manner as indicated by better clinical conditions, macroscopic aspects and only minor small intestinal mucosal changes with preserved epithelial lining due to PACAP prophylaxis. Our results are supported by two previous studies demonstrating that DSS-induced colitis was exacerbated in PACAP^−/−^ mice [8], [9], whereas the devastating phenotype could be rescued by intraperitoneal synthetic PACAP administration at day 1 following colitis induction [9]. In our study, the better outcome following prophylactic PACAP treatment was due to a dampened small intestinal Th1-type immunopathology as indicated by a reduced accumulation of T lymphocytes, the major driving forces of *T. gondii* induced ileitis, in the ileal mucosa and lower local expression of the key regulator IL-23p19 and pro-inflammatory mediators such as IFN-γ, nitric oxide, and the chemokine MCP-1. Previous *in vitro* studies revealed that PACAP inhibits the proliferation and migration of T lymphocytes and concomitant Th1 cytokine release [3], [34], and further acts as a “macrophages inactivating factor” [35] given that pro-inflammatory cytokine release by stimulated macrophages was reduced due to PACAP action [36]. In line with this, numbers of recruited neutrophils, monocytes, and macrophages were reduced in PACAP-treated mice in our study, which in turn reduced oxidative stress to the intestinal epithelium. Our data are further supported by a study demonstrating a diminished influx of lymphocytes, neutrophils, and macrophages into the peritoneal cavity of PACAP-treated mice suffering from acute peritonitis [37]. Furthermore, prophylactically PACAP-treated mice displayed higher numbers of ileal FOXP3+ Tregs as compared to control mice at day 7 p.i. In support of this, PACAP^−/−^ mice exhibited reduced numbers of proliferating Tregs in experimental autoimmune encephalomyelitis [38], [39]. Interestingly, VIP and PACAP have been previously shown to generate tolerogenic dendritic cells (DCs) subsequently inducing functional Tregs *in vitro* and *in vivo* for maintaining peripheral tolerance [40]–[42]. Hence, it is tempting to speculate that the observed increase in ileal Tregs in prophylactically with PACAP treated mice at day 7 p.i. was most likely due to the PACAP-induced “tolerogenic DC – Treg axis” which might act as one of the potential compensatory mechanisms to combat the parasitic infection. In our study the dampened intestinal pro-inflammatory responses exerted by PACAP prophylactic treatment during acute ileitis were accompanied by an up-regulated expression of the anti-inflammatory Th2-type cytokine IL-4 in MLNs. In line with this, *in vitro* studies revealed that macrophages treated with PACAP gained the ability to induce Th2-type cytokines such as IL-4 and to inhibit Th1-type cytokine expression levels in primed CD4+ T cells [43]. When antigen-immunized mice were challenged with PACAP, decreased numbers of IFN-γ producing cells could be observed, whereas, conversely, IL-4 secreting cells increased [43].

Remarkably, PACAP-induced anti-inflammatory effects were not restricted to the small intestine in our study, but could also be observed at extra-intestinal locations such as liver, kidneys, and lungs. *T. gondii* infected mice suffering from acute ileitis were previously shown to display an increased hepatic abundance of activated CD4+ T cells [21] and increased levels of pro-inflammatory cytokines such as TNF-α, IFN-γ, MCP-1, IL-6 in the liver as shown in our very recent [44]) and actual study. However, histopathological changes in lungs and kidneys of *T. gondii* infected mice with acute ileitis have not been reported yet. Seven days following *T. gondii* infection, peri-bronchial cuffs consisting of leukocytes with hemosiderin deposits therein (most likely due to hemosiderin-laden macrophages) could be observed in lungs, whereas in kidneys of diseased mice glomeruli were pyknotic, degenerating and apoptotic and the Bowman capsule enlarged indicative for severe renal dysfunction. It is well known that during acute ileitis viable commensal intestinal bacterial species such as *E. coli* can translocate through the compromised epithelial cell barrier into the intestinal lamina propria, and subsequently come in contact with immune cells thereby further exacerbating the inflammatory scenario [27], [45]. When translocated bacteria or their cell wall constituents such as LPS reach the blood stream, sepsis and subsequent multiple organ failure with fatal consequences occur [27]. Hence, it is remarkable that following PACAP prophylaxis the observed histopathological changes leading to organ failure could be prevented in our study. The extra-intestinal immune-modulatory properties of PACAP were characterized by lower extra-intestinal and systemic expression of pro-inflammatory cytokines whereas anti-inflammatory cytokines such as IL-4 in liver and systemic IL-10 levels measured in serum and spleen were higher in mice subjected to PACAP prophylaxis. Previous *in vitro* studies revealed that PACAP inhibits the production of pro-inflammatory mediators by LPS-stimulated macrophages, whereas IL-10 release was enforced [43], [46], [47]. In line with our extra-intestinal results demonstrating ameliorated hepatic, renal and pulmonal inflammatory responses in *T. gondii* infected mice with prophylactic PACAP treatment, PACAP has been shown to protect against renal damage in different renal injury models [47]–[51] and against murine liver ischemia and oxidative stress [51], [52]. Furthermore, PACAP exerts significant anti-inflammatory effects in endotoxin-induced acute pulmonal inflammation [53]. Due to the anti-inflammatory and broncho-relaxant properties, synthetic PACAP analogues have been developed for treatment of bronchial asthma [53], [54]. Moreover, PACAP has been shown to inhibit TLR-4 activation during experimental traumatic brain injury [55]. Given that the immunopathology underlying acute *T. gondii* induced ileitis is initiated and perpetuated by bacterial LPS via TLR-4-dependent signaling [22], [23], dampening of TLR-4 dependent signaling reveals an important key stone of mechanism by which PACAP exerts its multi-facetted beneficial effect. In line with this, PACAP proved effective in preventing experimental endotoxin sepsis and shock [47], [56], [57].

It is noteworthy that 20% of mice even survived the acute disease for more than 4 weeks when treated prophylactically with PACAP, whereas all control animals had died by day 9.5 p.i. But that time, however, 40% and 80% of mice from the therapeutic and prophylactic PACAP treatment groups, respectively, were still alive. The long-term survival effect upon PACAP prophylaxis is remarkable given that the duration of prophylactic treatment was relatively short since the synthetic compound was applied for only six days in total and withdrawn by day 7 p.i. Furthermore, the biological impact of PACAP-induced beneficial effects becomes more plausible in such a devastating model. It is tempting to speculate that the survival rates would have been even higher if PACAP had been administered further on and not withdrawn by day 6 p.i. One should take into account that when considering a synthetic compound for combating systemic inflammatory disease in humans (irrespective of its etiology), not a single molecule but rather a plethora of molecules involved in the signaling cascade of the hyper-inflammatory scenario should be targeted [47]. Hence, PACAP seems a promising candidate in this regard.

In conclusion, synthetic PACAP ameliorates intestinal and systemic inflammation following *T. gondii* induced acute ileitis in a duration-of-treatment dependent manner and hence provides an alternative option for prophylaxis and treatment of intestinal inflammation such as inflammatory bowel diseases.

## Supporting Information

Figure S1 **Comparable ileal** ***T. gondii*** **DNA loads in PACAP and placebo treated mice.** Ileitis was induced by peroral infection of mice with *T. gondii* at day 0. Parasitic DNA levels were determined in ileal *ex vivo* biopsies following PACAP prophylactic treatment (Proph, white bars), PACAP therapy (Therap, gray bars) or placebo application (black bar) at day 7 post infection by quantitative real time PCR. Numbers of analyzed animals are given in parentheses. Mean values and standard errors of the mean are indicated. Data are pooled from three independent experiments.
(TIFF)
