# Supplementary figures and images for: Pituitary Adenylate Cyclase-Activating Polypeptide Ameliorates Experimental Acute Ileitis and Extra-Intestinal Sequelae
Source: PLoS One. 2014 Sep 19;9(9):e108389. doi: 10.1371/journal.pone.0108389 (PMC4169633; doi:10.1371/journal.pone.0108389)

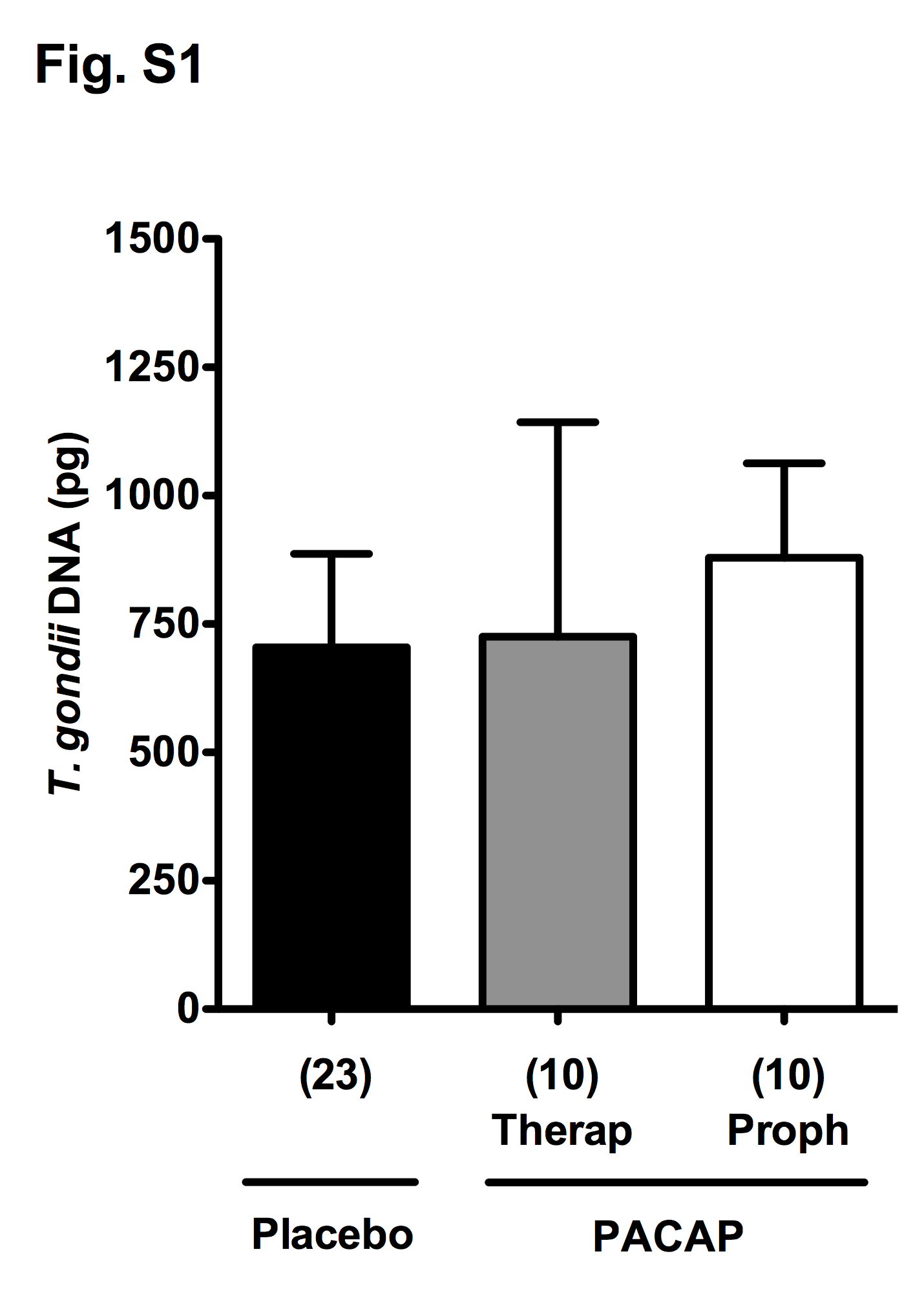

Supplement: Figure S1 — Comparable ileal T. gondii DNA loads in PACAP and placebo treated mice. Ileitis was induced by peroral infection of mice with T. gondii at day 0. Parasitic DNA levels were determined in ileal ex vivo biopsies following PACAP prophylactic treatment (Proph, white bars), PACAP therapy (Therap, gray bars) or placebo application (black bar) at day 7 post infection by quantitative real time PCR. Numbers of analyzed animals are given in parentheses. Mean values and standard errors of the mean are indicated. Data are pooled from three independent experiments. (TIFF) [file pone.0108389.s001.tiff]
